# Alternate Antimicrobial Therapies and Their Companion Tests

**DOI:** 10.3390/diagnostics13152490

**Published:** 2023-07-26

**Authors:** Sriram Kalpana, Wan-Ying Lin, Yu-Chiang Wang, Yiwen Fu, Hsin-Yao Wang

**Affiliations:** 1 Department of Laboratory Medicine, Linkou Chang Gung Memorial Hospital, Taoyuan 333423, Taiwan; vbkk2005@gmail.com; 2 Department of Medicine, University of California San Diego, San Diego, CA 92093, USA; wal018@health.ucsd.edu; 3 Department of Medicine, Harvard Medical School, Boston, MA 02115, USA; ywang134@bwh.harvard.edu; 4 Department of Medicine, Brigham and Women’s Hospital, Boston, MA 02115, USA; 5 Department of Medicine, Kaiser Permanente Santa Clara Medical Center, Santa Clara, CA 95051, USA; yf3255@gmail.com

**Keywords:** antimicrobial resistance, antimicrobial peptides, aptamers, companion diagnostics, bacteriophages, companion diagnostics

## Abstract

New antimicrobial approaches are essential to counter antimicrobial resistance. The drug development pipeline is exhausted with the emergence of resistance, resulting in unsuccessful trials. The lack of an effective drug developed from the conventional drug portfolio has mandated the introspection into the list of potentially effective unconventional alternate antimicrobial molecules. Alternate therapies with clinically explicable forms include monoclonal antibodies, antimicrobial peptides, aptamers, and phages. Clinical diagnostics optimize the drug delivery. In the era of diagnostic-based applications, it is logical to draw diagnostic-based treatment for infectious diseases. Selection criteria of alternate therapeutics in infectious diseases include detection, monitoring of response, and resistance mechanism identification. Integrating these diagnostic applications is disruptive to the traditional therapeutic development. The challenges and mitigation methods need to be noted. Applying the goals of clinical pharmacokinetics that include enhancing efficacy and decreasing toxicity of drug therapy, this review analyses the strong correlation of alternate antimicrobial therapeutics in infectious diseases. The relationship between drug concentration and the resulting effect defined by the pharmacodynamic parameters are also analyzed. This review analyzes the perspectives of aligning diagnostic initiatives with the use of alternate therapeutics, with a particular focus on companion diagnostic applications in infectious diseases.

## 1. Antimicrobial Resistance: The Current Scenario

Antimicrobial resistance is a global health problem warranting maintenance of balance between initiation and cessation of antimicrobial treatment. A better usage of antibiotics is addressed by avoiding misuse or its overuse. Misuse of antibiotics results from diagnostic errors either from an inappropriate test or delayed test. The antimicrobial stewardship programs pave the way in preventing the diagnostic errors by improvising the protocols, to reduce drug related-resistance, adverse events, and reducing the cost at the socio-economic level. The evolution of resistance cannot be slowed, but could be addressed by avoiding inappropriate use, or by formulating optimal and appropriate drugs [1]. Other factors contributing to antibiotic resistance are lack of hygiene and infection prevention control [2].

Drug development is always in a race with evolution of resistance. As with any other drug development, new antimicrobial agents should comply with the speed of screening, patent protection, approval process, and marketing [3]. Both drug development and delayed evolution of resistance are not mutually exclusive, but either or both of the two may be inherently more effective. Studies have, however, pointed out that slowing of evolution is the only option, because effective drug development is nearly impossible. This review describes the alternate therapies, including monoclonal antibodies, phage therapy, aptamers, and antimicrobial peptides, involved in infectious disease and the applicability of the associated companion test (CDx).

## 2. Diagnostic Stewardship as a Leverage in Antimicrobial Resistance

Diagnostic stewardship is “the right tests for the right patient at the right time for an optimal clinical care”. It encompasses reducing diagnostic errors and is integrated to antibiotic stewardship [4]. The intervention may occur at “preanalytical,” or “analytical”, or “postanalytical”—similar to clinical decision support tools [5].

The goal in infectious diseases is proper diagnosis of infection, source location, and organism eradication. The diagnostics have evolved with advancements in chemistry, immunology, molecular biology, biomedical engineering, and genomics. Automated and multiplexed detection is helpful in pathogen identification from different sources such as blood, urine, tissue, sputum, cerebrospinal fluid, respiratory secretions, and stool samples [6].

Diagnostic stewardship includes diagnostic pathway and intervention points that involve multidisciplinary collaboration, in which a key diagnostic test should be considered for diagnostic stewardship. Diagnostic stewardship is established under a comprehensive antimicrobial stewardship program. It also includes research on the right tests and clinical outcomes needed, rather than comparison with other tests. In addition, to the importance of sensitivity and specificity, it also controls the cost, by avoiding unnecessary tests. With low pretest probability, test results pose challenges leading to overtreatment, but is exaggerated by the high sensitivity of new techniques. To steer through these challenges, diagnostic stewardship improves the ordering, collection, processing, and reporting of diagnostic tests for a better patient management ensuring “the right test on the right patient at the right time” [4].

## 3. Drug Target

The introduction of antibiotic drugs and the evolution of resistance have time and again been reported as the ‘drug resistance treadmill’ [7]. The development of new antibiotics generally comes with failure rates of 95%, further stressing the difficulties in this area. Economically, newer antibiotics are costly and may affect profits from the older but effective antibiotics; newer antibiotics are stewarded as a last resort, therefore, resulting in less sales. The WHO reports that with 42 antibiotics under clinical development, only 11 can potentially be used in treatment [8]. Since the emergence of resistance is outpacing drug discovery, the development of new alternate forms of drugs is the only option. Validated drug targets are necessary to improve the therapeutic response to infectious disease. Several molecular targets prevail in identification, and validation is challenging. To achieve the most successful treatment of infectious diseases, it is essential to involve multi-omics approaches (genomics, transcriptomics, proteomics, and metabolomics) to substantially validate the drug targets.

### 3.1. The Drug Portfolio

Accordingly, a “drug” in a whole drug therapy comprises one or more active ingredients. The “drug portfolio” is a collection of approved drugs effective against a disease. In the case of antibiotics, at any given time, a single drug is actively continued until resistance appears, reaches threshold frequency in the population (2010), and is eventually discontinued. A drug from the drug portfolio, if available, replaces the resistant drug [1]. Simultaneously, new drugs are added to the drug portfolio depending on the stages of development. Multiple drugs are used simultaneously, even though a drug is not abandoned [9].

The agar overlay process, namely, the Waksman platform, has been the antibiotic discovery platform since 1937, identifying many antibiotics currently in clinical use. The details of the golden era of antibiotic drug discovery have been reviewed in detail [10]. Succeeding the Waksman platform, antibacterial semi-synthetics with modification to the existing scaffolds were developed. The modifications provided chemically stable molecules with reduced side effects. Resistance to semi-synthetic antibacterial has also evolved rapidly [11]. Semi-synthetic antibiotics with chemical modification saw the dawn of the medicinal chemistry era, which, along with the Waksman platform, yielded clinically relevant antibiotics with significant potency and lesser side effects.

The antibiotic discovery platforms (ADPs) is the list of exhausted drugs, with redundant discoveries and/or failed clinical translations. Statistically, between 2004–2009, the overall rate of antibacterial approval was a single drug per year [12], which doubled between 2011–2014 [13], but has improved since 2014. The trend is improving, with an increase in the number of drug runners in the pipeline but a lower approval rate [14]. In the present scenario, it is necessary to look beyond conventional antibiotics, the classical therapy. Alternate forms are non-classical therapeutics such as antibodies, antimicrobial peptides, bacteriophages, anti-virulence strategies, vaccines, immune stimulants, and antibiofilm agents that are currently being evaluated for their efficacy [15]. Phage therapy began two decades prior to the first clinical antibiotic, but was banished in the 1940s. Broad-spectrum antibiotics were considered the “wonder drugs” in clinical settings. The major events in phage therapy, including the research and development timeline, have been reviewed by [16]. Phages are highly specific for their hosts and evolve over time [17]. The direct effect of phage in the pathogenic bacteria is advantageous over the collateral damage induced by the antibiotic [18].

### 3.2. Non-Classical Antimicrobial Therapies

The history of antibiotics is cyclical. Lack of judicious use of the existing drugs combined with an unfruitful antibiotic development has diverted the search for non-classical therapeutic options [15,19]. A potential alternative to antibiotics includes phage lysins and probiotics as therapeutics, and antibodies and vaccines as prophylactics. Alternatives to antibiotics act via the immune system, which necessitates the use of non-human primates [15].

#### 3.2.1. Monoclonal Antibodies—The On-Target Molecule

Antibacterial monoclonal antibodies (mAb)s are interesting therapeutic options. Pharmacodynamic mechanisms are distinct from other antimicrobial agents. Firstly, the high specificity of mAbs does not affect the normal microflora, thereby reducing the burden for cross-resistance. The pharmacokinetics (PK) of mAbs is defined by target-mediated drug disposition (TMDD) due to opsonophagocytosis or the formation of antibody–toxin complexes. Accordingly, high-affinity drug binding to its target affects the PK [20]. With lower concentration, high-affinity mAb–target binding results in an accumulation of the drug at the sites of action, resulting in a large apparent volume of distribution of mAb. On the other hand, at a higher concentration, the target sites are saturated, and the tissue: plasma mAb concentration ratios increase, resulting in the decrease in the apparent volume of distribution.

Further, the mAb TMDD mediates endocytosis, which results in lysosomal engulfment and degradation of the mAb–target complex, accelerating mAbs clearance. This shortens the biological persistence, reduces half-life, and decreases dosage repetition. The antibacterial mAbs currently developed exhibit TMDD characteristics, impacting efficacious dosing in nonlinear PK due to differences in bacterial burden and immune status [21].

Pharmacodynamically, the action of mAb depends on its isotype and structure, nature of the target, and the pathogenesis. Anti-exotoxin mAbs attenuate bacterial pathogenesis including bacterial clearance by antibody-dependent phagocytosis, complement-mediated bactericidal activity, or immune-system-independent bacterial killing. Antimicrobials conjugated to mAbs stimulate immune effector functions. Bacterial pathogenesis orchestrated by toxins or by invasion are mediated by virulence factors that are conserved with a genus or a species. The virulence factors determine the site and the type of infection. For example, *E. coli* causes intestinal infections or extra intestinal infections [22] with different differentiated “pathotypes”, based on the type of disease they cause, and their virulence mechanisms (e.g., enterotoxigenic, enterohemorrhagic *E. coli*).

The invasion pathogenesis requires exotoxins or injector effector molecules that enable the bacterial fitness to promote rapid replication. Bacteria pathogenesis evades phagocytosis and complements mediated cell killing. Neutralization of toxins or the virulence factors by antibacterial mAbs is an approach that has been successful against tetanus, diphtheria, and botulism. Neutralization effects do not subject evolutionary pressure on bacteria, restricting the emergence of mutants. Cross-neutralizing mAb or the combination of mAbs with different specificities are desired for effective toxin neutralization. Although the smaller size fragments of Abs are advantageous, the lack of the Fc component of mAb impacts the half-life of mAbs [23].

Synergistic effect is expected when anti-toxin mAbs are used in conjunction with antibiotic therapy [24]. The majority of mAbs have a human IgG1 backbone with a kappa light-chain-mediating potent effector functions that includes complement fixation, complement dependent cytotoxicity (CDC), and opsonophagocytic killing (OPK). IgM forms have low affinity but are highly effective in complement-mediated killing and complement-dependent phagocytosis. Therapeutic mAbs with bactericidal activity are less likely to benefit immunocompromised patients due to low complement activity [25]. The mAbs of IgG isotypes are highly specific and dose-dependent with limited cross-reactivity, necessitating a wide range of unique mAbs. Hence, IgM and IgA isotypes are preferred for more cross-reactivity.

Three anti-bacterial mAb products have been approved for human use—raxibacumab (ABthrax^®^), obiltoxaximab (Anthim^®^), and bezlotoxumab (ZINPLAVA^TM^). The FDA-approved antibacterial mAbs are predominantly IgG isotypes of a molecular weight of around 150 kDa, with two antigen-binding domains and a highly conserved crystallizable region (Fc). Therapeutic mAbs exhibit a nonlinear PK, with AUC not proportional to the dose, as well as being dependent on bacteria load, its accessibility, affinity, and dosage. In the absence of mutation in mAbs, it is unlikely to apply the mutant selection window (MSW) in mAbs, which is discussed further in this review.

#### 3.2.2. Antimicrobial Peptides: Arming the Enemy

Antimicrobial peptides (AMPs) are amphipathic peptides with diverse composition and secondary structures that exert antimicrobial activities [26,27] (Guilhelmelli, Vilela et al., 2013). AMPs are bioactive and naturally a part of the biological defense system for pathogen inactivation. The mechanistic action of AMPs include disruption of bacterial cell membranes, modulation of host immune responses, and regulation of inflammation processes [28]. There are a plethora of unconventional sources of AMPs, including recombinant or synthetic AMPs that can be synthesized [29]. The effectiveness of AMPs is dictated by the dose, duration, route of application, formulation, target tissue, and host microbiota [30]. AMPs represent an apt choice in the development of new antibiofilm drugs that can potentially induce significant disruption of biofilm formation at different stages, including inhibition of adhesion, downregulation of quorum-sensing factors, and disruption of the pre-formed biofilm [31].

The pharmacodynamics of AMPs are different from the pharmacodynamics of antibiotics as their dose–response characteristics are different [32]. The dose–response curve has the Hill coefficient (κ), which is the slope of the curve; ψ_max_, which is the maximal bacterial growth rate in the absence of antimicrobial; and ψ_min_, meaning the minimal bacterial growth rate at high concentrations of antimicrobial and zMIC, which is the dynamic MIC [33]. The Hill coefficient (κ) is higher for AMP, therefore, producing a steep curve as compared to the curve for antibiotics [32]. The speed of killing targeted organisms is significantly higher for AMPs when compared to antibiotics [34,35].

AMPs display a narrower MSW; hence, they are less likely to evolve and develop resistance when compared to antibiotics [35]. Bacterial resistance evolution against AMPs is highly unlikely, as shown in previous studies [36], although in vitro experiments have demonstrated the evolution of resistance to AMPs [37] and characterized their resistance mechanisms [38]. Although in vitro resistance to AMPs evolves readily, it is uncommon in vivo [39].

Studies have also identified the evolution of κ, ψmin, ψmax, and the influence of cross-resistance or cross-sensitivity. An increase in κ and ψ_max_ reduces the probability of resistance development. AMPs display similar properties, with a smaller MSW [32]. A high Hill coefficient (*κ*) is a promising characteristic of new antimicrobials. The other feature of AMPs is mutagenesis and maximum effect rather than the size of the MSW [40]. At low concentrations, the emergence of resistance in AMPs takes longer than in antibiotics. Additionally, in MIC, it is more effective against organisms than antibiotics, given the quicker removal of sensitive strains due to AMPs’ high *κ* and low ψ_min_.

Combination of AMPs results in increased *κ* values, which slows the evolution of resistance [32]. Resistance increases the MIC for antibiotics by 2–3 orders of magnitude in a relatively small bacterial population [41], but that has not been observed for AMPs. Though AMPs provide promising leads for drug development [15], their conserved killing mechanisms also up for debate.

#### 3.2.3. Aptamers—Emerging Therapeutics

Aptamer are single-strand DNA or RNA oligonucleotides of 5−25 kDa, with high affinities and specifically binding to proteins or nucleotides [42]. Aptamers are flexible but stable, and capable of accessing internal epitopes while retaining their primary conformation even after denaturation [43]. Their functions are diverse, which makes them favorable as therapeutic agents, especially the ability to act as carrier molecules [44]. Primarily, aptamers are non-toxic, less immunogenic, with a well-defined 3D structure against toxic substances, and targets in complex mixtures [45,46,47,48]. However, aptamers are susceptible to nuclease activity (Lee, Yigit et al., 2010) and subject to renal filtration [49].

Mechanistic and structural factors trigger the affinity and the dissociation of aptamers [50]. Aptamers’ binding affinities are comparable to antibodies’, with dissociation constants (K_d_) ranging from low picomolar to mid-nanomolar, mostly <10 nmol/L [50]. Therapeutic purposes are the same as mAbs, with no requirement of any organisms for the in vitro selection. Theoretically, aptamers can be targeted for intracellular, extracellular, or cell-surface components, including proteins. They are therapeutically used to block protein–protein interactions. Aptamers form well-folded stable secondary and tertiary structures that greatly affect the binding affinity. For example, aptamer affinity can be altered by modifying the 2′-hydroxyl position with a 3′-endo sugar pucker. Forming pseudoknot structures can also impose high affinities [51].

Kinetically, a low K_d_ causes tighter binding to the target molecule, leading to a higher degree of geometric interaction and complementarity. Aptamer-based “antibiotic” approach masks the bacteria to evolve resistance, including the complement system (Barnes and Weiss 2001). Clinically relevant aptamers against bacterial infections include DNA aptamers (Lyd-1, Lyd-2, Lyd-3) against *S. pneumoniae* [52,53,54]. Among them, Lyd-3 is an effective anti-biofilm aptamer often used in combination with antibiotics to prevent bacterial colonization [55].

Aptamers are significantly inhibitory against bacterial toxins—including Staphylotoxin A, anthrax toxin, and botulinum toxin [52]. Aptamers AR-27, AR-33, AR-36, and AR-49 directly act against bacterial toxins, particularly the α-toxin of *S. aureus* [56]. Aptamers bind to bacterial lipopolysaccharide with high affinity and conjugate to classical complement systems (C1qrs), triggering the destruction of bacteria beyond levels attributable to non-specific LPS-induced activation of the alternate pathway. Aptamers also exert a synergistic effect with antibiotics or nanoparticles [57]. In vitro experiments with aptamer–single wall nano tubes (SWNTs) inhibited ~36% biofilm formation than SWNTs alone. Aptamer–ciprofloxacin–SWNTs had a higher anti-biofilm efficiency than either component, suggesting a new strategy to control biofilms [58].

The identification of binding sites in DNA sequences is important in determining their functions. The systematic evolution of ligands by exponential enrichment (SELEX) is one approach to select from a oligonucleotide library, which is used in therapeutic applications. For antagonistic effect, the aptamers dock with the target to prolong the effects. Aptamers with biological relevance are optimized to have high affinity, specificity, and half-life SELEX protocols are able to increase aptamers’ target affinity (by decreasing off-rates) and specificity [59,60]. Compared to unmodified SELEX libraries, only modified or slow off-rate modified aptamer (SOMAmer) libraries isolate high-affinity aptamers. To minimize nonspecific binding of SOMAmers, aptamers with dissociation rates >30 min were selected [61].

#### 3.2.4. Phage Therapy—The Predator-Prey Re-Visited

Phage therapy is a potential alternative to antibiotic therapy, and its resurgence has been supported by theoretical views [62]. Monophage therapy is hampered by the emergence of bacterial phage resistance [63]. The shortcomings of monophage therapy are overcome by the phage cocktails—the polyphage. Phage cocktails can be designed to target a single bacterial strain, multiple strains of a single bacterial species, or multiple species with a reduced predictability of phage pharmacokinetic and pharmacodynamic properties [64].

The combination of monophage and polyphage therapies sequentially as an alternative to simultaneous administration of phage cocktails is effective [65]. Phage production is increased in the presence of sublethal concentrations of certain antibiotics, a phenomenon named as phage antibiotic synergy (PAS), and reduces the development of resistance [66]. In combination with antibiotics and applying the “order effect”, using phage therapy prior to antibiotics achieves maximum killing. Thus, the optimization of the time of administration of combinational therapy can potentiate the efficacy of phage [67,68]. The expansion of PAS also limits antibiotic usage, which is considered as the second wind against MDR pathogens [69].

The pharmacokinetic determinant of phage therapy is the phage dose, defined as the number of phage particles to be administered. Visible plaque enumeration on agar plates does not reflect total phage particles. Phage counts are expressed as efficiency of plating, which is the ratio of the plaque-forming unit (pfu) of phages on the target bacterial strain relative to that on the reference strain [70]. Quantitative PCR is performed to assess the viral load kinetics [71]. With regard to pharmacodynamics, phage activity is assessed as the efficiency of plating in agar using direct spot test, with different coverage of *E. coli* and the antibacterial killing in a broth as planktonic killing assays [72]. Hence, an understanding of phage pharmacokinetics/pharmacodynamics (PK/PD) will substantially optimize phage therapy [73].

The other major PK/PD obstacle is the biofilm that is overcome by phages [74]. The ‘predator–prey’ relationship between phages and susceptible bacteria inducing self-replication is not an independent variable as in the exposure–response relationship noted in antibiotic treatment, therefore, it cannot be quantitated in terms of PK/PD index approach [75]. The PAS represents synergistic effect [76] and its mathematical model provides an understanding of phage–host interaction [76], deriving the proliferation threshold and inundative threshold [77]. Theoretical understanding of bactericidal dosing consequences is calculated at a minimum phage density (i.e., MBCt).

**Figure 1:**
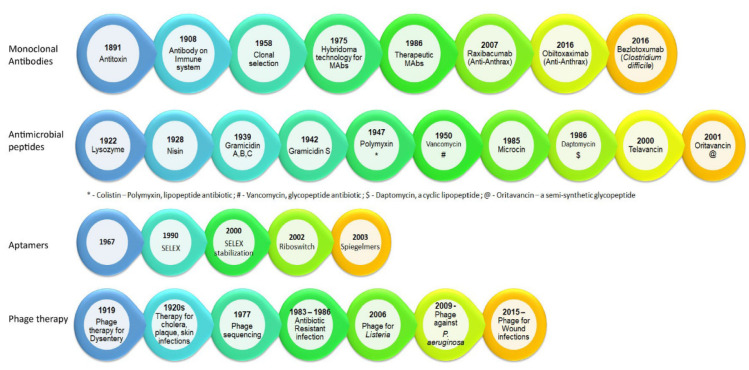
Timeline of alternate antimicrobial therapies.

The history of discovery and time of approval of antimicrobial therapies (Figure 1) highlight phage therapy as a forerunner of other alternate therapies and a comparative analysis shows the four alternative therapies that have a synergistic effect to antibiotic treatment (Table 1).

## 4. The Target—What Is Expected?

Personalized medicine in infectious diseases is an emerging concept involving rapid, accurate, and comprehensive diagnostic microbiology assays [81]. Various nucleic acid testing assays for the detection of resistant bacteria from clinical settings have been formulated. Comparative genomics utilizing bioinformatic and microarray technology aids in the identification of virulence determinants, antimicrobial drug targets, vaccine targets, and new markers for diagnostics. Comparative genomics has been assumed by pharmacogenomics for predicting adverse effects caused by the therapeutics.

## 5. Phenotypic Tests

### 5.1. Standard Antimicrobial Susceptibility Testing

Antimicrobial susceptibility testing (AST) results are crucial for initiating proper treatment. The clinical breakpoint of antimicrobial concentration is assessed from its in vitro efficacy, pharmacokinetic/pharmacodynamic (PK/PD) parameters in animals, and the establishment of efficacy/toxicity in a pathogen-induced animal model. Accordingly, when correlating the clinical outcome with in vitro susceptibility tests, “the 90-60 rule” is applicable, in which 90–95% of susceptible patients and 60% of resistant patients respond to therapy [27]. However, the recommended doses of antibiotic have failed in clearing the infection in 5–10% cases [82].

As with other antimicrobial drug-related conditions, AST methods for AMP pre-clinical development need to be devised. AMP-specific endpoints do not equate to 100% growth inhibition. The AST for AMP is broth microdilution [68,83]. The agar method is avoided, as positively charged AMP interact with negatively charged components in agar and neutralize their activities [84,85]. This AST method needs to be calibrated to the ISO standards for an accurate, robust, reproducible, clinically valid method that is amenable to automation.

#### 5.1.1. Minimum Inhibitory Concentration

Antimicrobial resistance is assessed in terms of concentration-dependent criterion such as minimum inhibitory concentration (MIC), mutation prevention concentration (MPC), and mutant selection window (MSW). The antibiotic selection gradients were developed in vitro in 1997 (Baquero and Negri 1997). The selection for antibiotic resistance is connected at the subcellular and supracellular level, demanding frequent monitoring for changes (Baquero 2011). By definition, MIC is the lowest drug concentration inhibiting or blocking the growth of 10^5^ colony=forming units/mL (CFU/mL) of the bacterium. Methods for MIC testing include broth microdilution, agar dilution, or the E-test. In broth microdilution testing, the drug is added to the medium at desired concentration and incubated for 18–24 h in a 96-well microplate. In this assay, the lowest drug concentration preventing visible growth is recorded as the MIC. In agar dilution testing, the bacteria are inoculated in agar plates, with the drug incorporated at pre-determined concentrations directly, and the lowest growth inhibitory drug concentration is the MIC. In the E-test, the entire agar plate is inoculated, and the E-test strip containing gradient drug concentrations is added on the surface, and the point on the E-test strip that intersects the line of bacterial inhibition after intubation is recorded as the MIC.

There must be a comparison of MIC with previously established reports of antimicrobial sensitivity. When the MIC recorded is below the susceptibility breakpoint, the bacteria is considered susceptible, whereas in the case that it is above the susceptibility breakpoint, the bacteria is non-susceptible or resistant. For in vitro susceptibility testing, 10^5^ CFU/mL is the standard and is relevant for clinical management. Antibiotic gradients ensure selection of bacteria with very small differences in MIC values [86]. In vivo confirmation of this principle of concentration-dependent selection was obtained in animal models and supported by mathematical modeling [87].

#### 5.1.2. Mutation Prevention Concentration and Mutant Selection Window

An in vitro susceptibility test that measures the probability of mutant subpopulations in the high density of bacterial population is the mutant prevention concentration (MPC) [88]. The MPC is a more advanced test, and is defined as the MIC of the most resistant cell present in the population. MPC applies only to susceptible organisms recommended by susceptibility criteria and breakpoints. Therefore, establishing the MPC may be of use to prevent resistance clinically [89].

In general, both susceptible and mutant bacteria are inhibited at drug concentrations exceeding the MPC, but not at concentrations below the MIC. The selection of antibiotic-resistant mutant bacteria is proposed to occur in a range of drug concentration that extends from the MIC of susceptible cells to the MIC of the least susceptible, which is the MPC that is defined as the MSW. Dosing to achieve drug concentrations more than the MPC likely blocks susceptible and mutant cell growth.

The MSW surpasses the MPC concept, and serves as an in vitro framework for identifying antimicrobial pathogens that lead to genetic resistance, and it validates the addition of a compound to combination treatment as it provides a validation on determining a compound to be employed as a part of a combination [90,91]. MIC is a hybrid and contextual pharmacodynamic variable, and is influenced by the test medium [90]. The standard test employs Mueller–Hinton broth (MHB) for rapid growth of bacteria, which is not the case in vivo. As the in vitro MIC and effective in vivo plasma concentrations differ, MICs differ between drugs, therefore, exposure to MIC results in the emergence of resistant mutations as well an accumulation of drug residues, which causes cell damage [92]. Further, MICs do not reflect dynamic antibacterial activities such as changes in kill rate and growth rate. The discrepancies between the in vitro and in vivo MSWs is overcome by the broth dilution method [93]. 

Another resistance-related parameter is the frequency of spontaneous mutant selection (FSMS) required for subsequent in vivo studies [94]. Single-step mutation is prevalent in bacteria, but the frequency of this spontaneous mutation is very low. More generally, the emergence of mutants is effectively combated by the host immune systems [95]. Mutants with a low FSMS value of 1 × 10^−6^ to 1 × 10^−8^ are effectively controlled by the host organism. The frequency of 1 × 10^−8^ is the threshold for reduced mutant selection in in vitro analysis [96].

The MPC is the obtained from the growth of no mutants in a minimal antibiotic concentration with a large number of bacteria (>10^10^) in agar [88]. The number of cells is represented as MPC_10_10. Mutants usually arise in the sub-MIC of antibiotics [97]. The MPC/MIC ratio ranges from 4 to >32, depending on the antibiotic and the strain [98]. In treatment, there is an abundance of selected mutants at antibiotic concentrations within the MSW, which increases the loss of its activity [95]. Currently, MPC-based resistance-restricting dosing schemes are not in application. Further studies regarding certain strains of bacterium and drug combinations have been reported for the reproducibility of MPC results [99].

The mutants are classified into two categories, dominant mutants and inferior mutants. Krajewska et al., (2023) proposed an in vitro determination of the resistance with a new broth dilution method, with proposed parameters including dominant MPC (MPC-D), inferior MPC (MPC-F), dominant MSW (MSW-D), and inferior MSW (MSW-F). MPC-D is the lowest drug concentration that prevents mutants selected from amongst 10^10^ CFU after 24 h of intubation for establishing a resistant population of at least 10 CFU/mL in a drug-supplemented broth culture with a high frequency, without a resistance-associated fitness loss [100]. The MPC-F and MSW-F refer to mutants with impaired fitness that can be selected in vitro in concentrations above the MPC-D but cannot dominate the population in the broth culture. These mutants, usually, are not able to dominate in the broth culture and are less likely to appear in subsequent in vivo studies. In the process of drug selection, mutant domination is more important than mutant selection for making MPC-D based, resistance-restricting dosing regimens.

### 5.2. In Vivo Altered Susceptibility Test

This has necessitated the improvisation of predictive values of AST. Standard AST uses Mueller–Hinton broth, which supports optimal bacterial growth, although the efficacy observed is not reflected in true clinical environment [101]. Host environment alters growth and expression patterns of essential genes, which impacts the AST [102,103,104]. An altered AST assay, which incorporates media mimicking the host environment, is able to predict with better accuracy than the standard AST. This assay is referred to as an in vivo altered susceptibility test (IVAS), which has aided in the identification and screening of compounds, and has paved the way for the re-purposing of omitted antibiotics. The IVAS platform has physiological relevance including against ESKAPE pathogens [105]. For instance, *P. aeruginosa* expresses essential genes for survival differently when grown in minimal medium containing lung sputum from cystic fibrosis as compared to those grown in lysogeny broth (LB) [106]. *P. aeruginosa* grown in Rosewell Park Memorial Institute medium with 20% human serum and 5% MHB exhibited the resistome genes when compared to that grown in MHB alone [107].

### 5.3. Cross-Resistance Analysis

By other means, antibacterial drugs not suited for therapy also exist. They arise from cross-resistance, which is the positive correlation between resistance and different antibiotics resulting from the inherent bacterial attributes, for example, sharing common mechanism [108]. Cross-resistance between chemically dissimilar antibiotics also occurs from horizontal gene transfer, potentially involving genes for resistance to multiple antibiotics. Conversely, negative correlation of resistance to different antibiotics is cross-sensitivity, or collateral sensitivity arising from complex relationships of resistance among different antibiotics requiring a multivariate approach with multiple drug resistances as the dependent variable.

Cross-resistance platforms detect cross-resistance between established and novel antibacterial agents [108]. A cross-resistance detection platform is designed with an initial iteration of the platform with a Gram-positive bacterium, namely, *S. aureus*, SH1000. A defined resistance genotype is set in each strain by cloning resistance genes harboring the selectable marker, and the expression of cloned resistance determinants is propelled by a constitutive promoter. *Staphylococcal* resistance determinants require the induction of resistance phenotypes to manifest. A resistant phenotype existing in clinical strains mediates profound resistance, implying a sub-optimal drug of choice. This proposes to deselect the compounds followed by addition to the antibacterial drug discovery toolbox.

### 5.4. Synergy Analysis

The combination of two antimicrobial agents exerts a cumulative effect or a synergistic effect, which is greater than the sum of their activities when used individually. Synergy testing is a sophisticated technique measuring the cumulative efficacy of a combination of antimicrobials that are not measured dynamically by routine susceptibility testing methods. The synergy tests detect antimicrobial interactions and the antagonistic effects of the combinations of drugs. There are four primary methods by which synergy is tested. the most commonly used in vitro assay is agar diffusion assay in solid media; others include checkerboard (CBA) assay in liquid medium, multiple-combination bactericidal antimicrobial testing (MCBT), Epsilometer test (E-test), and time–kill curve assays.

The pairwise identification and quantification of drug synergistic interactions is a laborious and time-consuming assay. There is no true “gold standard” method defined for synergy testing, and the mathematical models developed also derive opposite conclusions [109]. Conventional AST predicts the therapeutic outcomes of monotherapy but fails to be relevant in pan-resistant organisms such as *B. cepacian*. In this case, the MCBT can be useful to test the susceptibility against numerous combinations of antibiotics, with the results being available within 48–72 h. [110,111].

### 5.5. Dereplication—Antibiotic Resistance Platform

A major exertion in antibiotic discovery is the frequent rekindling of known compounds, requiring laborious “dereplication” to identify novel chemical entities. The antibiotic dereplication and identification of antibiotic adjuvants uses the “antibiotic resistance platform” (ARP). It is a library of approximately 100 antibiotic-resistant genes individually cloned into *E. coli*. Antibiotic-producing microbe ferments on a solid medium secrete secondary metabolites that diffuse through the medium. After 6 days of fermentation, the microbial biomass is removed, and a thin agar overlay is added to enable the growth of the *E. coli* indicator strains. The panel of ARP strains is pinned onto the surface of the antibiotic and incubated overnight. Only strains containing resistance to a specific antibiotic grow on the surface, enabling rapid identification of the produced compound [112].

### 5.6. Biofilm Eradication Concentration

The evaluation of biofilm-associated infections involves standardized biofilm susceptibility testing assays with endpoint parameters, such as the minimal biofilm eradication concentration (MBEC), minimal biofilm inhibitory concentration (MBIC), and biofilm prevention concentration (BPC), which guide the treatment of associated infections [82]. Recalcitrance is defined as the survival and growth of bacteria at high antibiotic concentrations. Non-genetic mechanisms can drive the resistance evolution. Resistance in biofilm increases with the continuous exchange of planktonic cells in biofilms. Therefore, it is worth noting that the timing and frequency of dosing influences the dynamics [113,114].

The MICs from planktonic bacteria assays are ineffective on biofilms. Instead, MBIC is used, which means the lowest drug concentration when there is no time-dependent increase in the mean number of biofilm viable cells. The minimal biofilm eradication concentration (MBEC) is the lowest concentration of antibiotic required to eradicate the biofilm [115]. The minimal bactericidal concentration (MBC) is the planktonic minimal bactericidal concentration defined as the lowest concentration that kills 99.9% of the cells [116]. The biofilm prevention concentration (BPC) is the drug concentration to reduce the cell density of a planktonic culture to prevent biofilm formation [117].

The minimal selective concentration (MSC) defines the concentration above the MSC of an antibiotic that results in the enrichment of a resistant mutant over the susceptible strain, which is the concentration of an antibiotic where the fitness cost of resistance is balanced. The minimal selective concentration values do not differ between planktonic cultures and biofilms [118]. In biofilm growth, shifts and distortions to the MSW has been predicted [114]. A promising avenue is to investigate intrinsic heterogeneities of biofilms and hindrance in resistance evolution.

### 5.7. Phage Dosing Parameters

Phage therapy also relies on phage dosing that includes MIC known as the “inundation threshold”, and minimum bactericidal concentration or the “clearance threshold”. The “minimum inundatory threshold” is the analogue of MIC that inhibits the bacterial population growth [119]. Phage “clearance threshold” is the number of phages required to kill bacteria. Minimum bactericidal concentration (MBC) is phage titer that reduces the density of a bacterial population to zero [120]. Clearance threshold measures the bacteria replicating, whereas killing titer does not. Phage adsorption rates are generally faster than bacterial replication. Hence, phage dosing is calculated in terms of phage titers, adsorption rates, and exposure duration. Phage MOIs are calculated from known phage titers, assuming incomplete phage adsorption. The prediction of minimal therapeutic phage density assumes purely “passive” treatment, which means the phage density necessary to at least prevent bacterial cultures from growing. MIC requires a greater phage density if phage adsorption is slower or a smaller phage density if bacterial replication is slower [121].

Phages should reach a concentration to adequately inhibit bacterial growth analogous to MIC, referred to as the “inundation threshold” (IT)—the concentration of phage at which the rate of bacterial growth equals the rate of phage infection [122]. When the concentration of virulent phage is above IT, susceptible bacteria are suppressed depending on the rate of growth of bacteria and the adsorption rate of phage particles. However, it does not directly depend on the bacterial concentration. Low-dose phage therapy attaining a concentration above IT can be achieved by increasing the rate of phage replication, which is dependent on the concentration of the susceptible bacteria. If the concentration of bacteria is less, phages degrade without completing their life cycle. The dynamics of phage–bacterium interactions yields a “proliferation threshold” (PT)—the concentration of bacteria above which the total phage population increases, below which it decreases.

Prediction of treatment outcome and resistance is possible with a range of bioinformatics tools that use a statistical learning approach or machine learning algorithms [123]. The statistical learning approach relies on the direct correlation between the baseline microbial profile, the therapeutic decision, and the response to treatment. The susceptibility scores that are used for combination therapy take into account the resistance mutations and synergistic effect of individual drugs in the regimen [124]. Computer-assisted therapy reduces the complexity of treatment but requires wide sharing of both proteomic and genomic databases.

Common interchange of genomic and proteomic information, such as minimum information about a microarray experiment (MIAME), minimum information requested in the annotation of biochemical models (MIRIAM), and minimum information used to describe a proteomic experiment (MIAPE), have meant a push to integrate databases in the management of disease [41,125,126]. These formats enable unambiguous interpretation of results and aim to ensure that experimental results in genomics, proteomics, and metabolomics are deposited in public databases before publication, as is already established for nucleotides. The Pathogen Information Markup Language (PIML) has also been recently introduced to enhance the interoperability of microbiology datasets for pathogens with epidemic potential [127].

## 6. Genotypic Assay

### 6.1. Non-Sequencing Assay

Genomics-based approaches detect genes relevant to virulence and antibiotic susceptibility. Proteotyping determines clinical patterns that are actually expressed. A combination of genomic and proteotyping could curb the antibiotic resistance pandemic [128]. The shotgun proteotyping of microorganisms has a huge potential, as it achieves a high resolution of identification. Employing a break-then-sort, bottom-up strategy, in combination with separation techniques such as liquid chromatography coupled with an MS analyzer, is called “shotgun” proteomics. Matrix-assisted laser desorption ionization time-of-flight (MALDI-TOF) mass spectrometry (MS) has recently become a useful analytical approach for microbial identification from the presence or absence of specific peaks on MS spectra and predicting antibiotic-resistant strains [129].

Molecular signatures of the synergistic interaction between antimicrobials and proliferation are reflected by the isobaric tags for relative and absolute quantitation (iTRAQ). This has revealed multiple sites of action on the bacteria, consistent with phenotype, thus, justifying the applicability of proteomics in understanding the molecular mechanisms from differential expression of proteins, aiding in diagnosis and therapy [130].

Microbial typing identifies organisms within a species. Traditional methods include biotyping and antibiogram typing, which later progressed to PCR-based molecular methods and sequencing techniques. Genotypic methods are reliable and reproducible, with greater discriminatory powers than the phenotypic methods. The choice of method relies on many factors, including cost and the diagnostic performance [131]. Phenotypic methods reflect gene expression products, distinguished on the basis of phenotypic parameters such as biochemical reactions and serologic properties, which often do not truly reflect results and have inconsiderable diversity [132]. Non-molecular methods require “preliminary” knowledge of bacterial type, and those includes biotyping, serotyping, antibiogram-based typing, phage typing, and proteomics typing.

Molecular (geno)typing methods evaluate genetic variations that include the absence or presence of plasmids, number and positions of repetitive elements, and the precise nucleotide sequence. Molecular genotyping is significantly advantageous compared to phenotypic methods. Genotypic methods are classified as non-sequencing and sequencing. Non-sequencing methods are plasmid profile typing, pulse field gel electrophoresis, ribotyping, restriction fragment length polymorphism, random amplified polymorphic DNA, polymerase chain reaction—arbitrarily primed, enterobacterial repetitive intergenic consensus sequence (ERIC), the repetitive extragenic palindromic sequence (REP), BOX-A1R-based repetitive extragenic palindromic, variable number tandem repeat, multiple locus variable number of tandem repeats analysis, amplified fragment length polymorphism microarrays, PCR-melting profile, and optical mapping [131]. Molecular tests signal the expression of implicated genes, and, therefore, are both transcriptomics-based and proteomics-based tests. Transcriptomic approaches to AMR detection are hindered by low correlation with protein levels [133]. On the other hand, proteomic techniques provide the strongest molecular evidence of resistance, but there no whole proteome-based AMR exists that is equivalent to genomic and transcriptomic approach [134].

Transcriptomic-based analysis of two sepsis-associated phenotypes reveals that the genes comprising the sepsis response signature (SRS) demonstrate significant overlap between the two sources of infection and with trauma patients, with gene expression and SRS membership temporally changed [135,136]. Another study revealed the genetic markers contributing to the adaptation of *P. aeruginosa* to disinfectant, suggesting the need to monitor for the markers in the practice of disinfection [137].

### 6.2. Sequencing-Based Assay

Sequencing-based typing methods include whole-genome sequencing that have improved to next-generation sequencing that is able to provide massive details, and has become a method of choice for vaccine development and pathogen marker detection, in addition to pathogen typing. WGS data are the technological basis for single-locus sequence typing, multi-locus sequence typing, core genome MLST, whole-genome MLST, and single nucleotide polymorphism (SNP) detection. Typing for *S. aureus* relies on sequencing the X region of the protein A gene with SLST application (Staphylococcus protein A spa typing). The 16S rRNA is a universal target and most frequently employed. Of the other techniques, SNP has the highest discriminatory power, detecting all polymorphisms. Horizontal gene transfer reveals several SNPs but results in only one allele change [138].

## 7. Companion Diagnostics in Infectious Diseases

**Figure 2:**
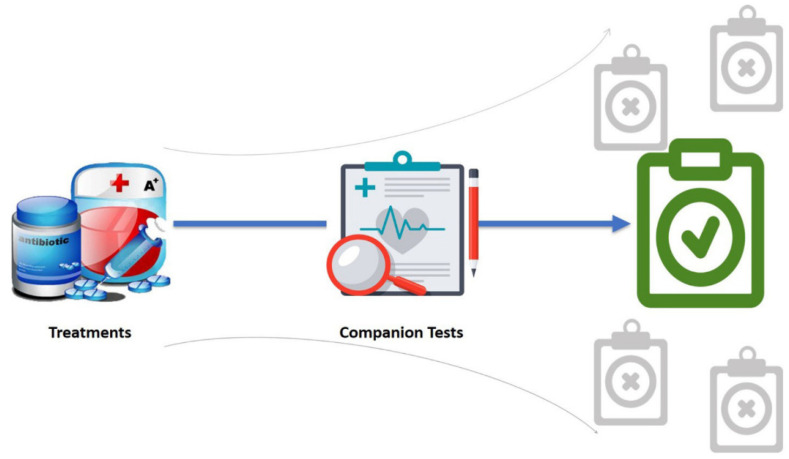
Illustration of the relationship between treatments and companion tests. Companion tests serve as a guide rail to link treatments to the corresponding targets precisely (green color). Without companion tests, treatments would fail due to being used in inappropriate or less susceptible subpopulations. To maximize the utility of treatments and minimize treatment failure, companion tests are the key to precise medicine.

Companion diagnostics (CDx) is a biomarker assay linked to a specific drug. It is a drug–diagnostic codeveloped model defining the probable benefit of a drug with a molecular diagnostic assay [139]. The CDx assay is an imperative decision tool for pharmacotherapeutic intervention, and an incorrect result may lead to inappropriate treatment. The assays have an essential role in the drug development process, and the efficiency depends on the performance. On approval of a drug, molecular diagnostic assays are regarded as a companion to the drug, and, hence, named companion diagnostics. CDx is important both in drug development and individualized treatment (Figure 2).

In the drug–diagnostic co-development for CDx assays, the critical factors are biomarker hypothesis, analytical validation, and clinical relevance. A biomarker hypothesis is developed from complete data on the pathophysiology and drug mechanism [140]. The selection of assay method depends on biomarker type. The assay sensitivity and specificity should be relevant to the sample [141]. With the development of prototypes for assays, analytical verification is performed to identify the stability, feasibility, and reproducibility. Then, the prototype is employed in an preliminary clinical trial to correlate the biomarker with clinical outcome. The outcome data are used to select the cutoff that defines the positive test result, later transforming as a decision tool. For example, trastuzumab (HERCEPTIN) improves survival in both adjuvant and metastatic HER2-positive breast cancer in patients [142]. Thus, the HER2 test became the first CDx, and additional assays were then approved by the FDA as CDx later. However, the HER2 assays were not codeveloped with trastuzumab, but were instead approved after the initial drug approval as technologies and commercial opportunities evolved [143]. Another example is the COBAS BRAF V600E test. The FDA approved this test along with vemurafenib (ZELBORAF) for metastatic melanoma, in which the overall survival was improved in patients with the BRAF V600E mutation compared to the control drug [144].

Plotting true positivity (sensitivity) against false positives (specificity) is the method used to determine the preliminary cutoffs. The area under the curve (AUC) is calculated for the different cutoff points and the one giving the largest AUC is selected as the final clinical cutoff for the assay, with values ranging between 0.50 and 1.0 (Nahm 2022). A value of 0.5 indicates no discrimination and 1.0 indicates perfect discrimination and a value of ≥0.8 suggests an excellent biomarker. The AUC of the ROC curve reflects the overall accuracy and separation performance of the biomarker (or biomarkers) and can be readily used to compare different biomarker combinations or models [145,146].

The analytical validation is complete with the selection of cutoff for the CDx. A CDx assay developed within a single laboratory is a laboratory-developed test (LDT). The final step is to determine the external reproducibility study among the clinical laboratories [147]. Clinical validation is not initiated before analytical validation including sensitivity, specificity, positive predictive value (PPV), and negative predictive value (NPV).

Clinical validation may include both randomized and nonrandomized trials in which the intrapopulation variability is decreased and the proportion of responsive patients increased by preselection [144]. The drug–diagnostic co-development is superior to the traditional all-comers approach [148]. Drugs in the pipelines are mostly developed from molecular subsets of patients relying on biomarker selection [149]. Traditional methods of drug development are replaced by adaptive development approaches that are efficacious in a relatively small, specific patient population. For instance, in the case of oncology drug development, whether or not they have a CDx assay linked to their use, there is a clear higher objective response rates for the group of drugs with a CDx from 80.2% to 41.0%, while for the group of drugs with no CDx assay linked to use, this ranges from 45.0% to 6.8% [140].

Antimicrobials are designed in an “one-size-fits-all” approach [150]. Clinical microbiology counts on the in vitro analysis to guide diagnosis and therapeutic efficacy [140,141]. The pharmacodynamic mechanisms are distinct for each of the antimicrobial agents. Since the 1950s, there has been a decline in drug introduction into the market, with a success rate of only 3% derived from conventional methods. However, genetic-based authorization is needed for 50% of all marketed drugs to be efficacious. The advantage of CDx is that it improves prognosis, predictive response and tolerance to treatment [147]. However, no specific CDx prescribed for infectious diseases have been approved by the FDA.

As mentioned, the principal objectives of CDx are the identification of appropriate patient groups, therapeutic products, prediction of adverse reactions, monitoring of response, adjusting the dosage scheme, and ensuring improved treatment outcomes [151]. This coordination of drugs and CDx in infectious diseases needs to be further addressed. Primarily, the important facets to be considered in devising the strategy for CDx are the analyte and the assay, the drug and the target. The nature of the antimicrobial agent to be employed and the pathogen against which it is targeted are fundamental in the CDx paradigm. With an expansion in the spectrum of antimicrobial agents, there is an unprecedented array for potential drug selection. Protocols need to be implemented to ensure specificity of therapies and their effects on direct and collateral targets. The drug-and-target-focused approach is envisioned to be braced by artificial intelligence and machine learning technologies.

The CDx assays for infectious diseases offer a wider range of in vitro assays and platforms with precision that allow for personalization of medicine (Table 2). The next-generation CDx assays are likely to be screening assays or confirmatory assays resulting in the gradual replacement of traditional approaches that are aimed at characterizing the mutational profile and proteomic component. The assimilation of diagnostic data by the state-of-art techniques, biomarker-driven analysis, and antimicrobial agents excluding antibiotics to the existing drug portfolio outlines a new horizon of CDx in addressing antimicrobial resistance. Analytical platforms for diagnostic assays are immunohistochemistry (IHC), quantitative polymerase chain reaction (qPCR), next-generation sequencing, imaging, and possibly immunoassays. Multiplexed and high-throughput mass-spectrometry-based platforms are preferred for the accuracy and rapidity required for CDx [139,152]. The translation of these identifications as biomarkers needs to be validated for disease in an affordable range.

A common barrier in the implementation of CDx clinically is the delay in adoption of assays due to lack of awareness of clinical relevance. Other factors in play are accessibility, availability, quality of sampling, and inaccuracy from an insufficient amount of data, and low quality. Lack of testing in the existing labs, and false negative and false positive reporting may also mislead treatment decisions. Further, the accuracy of tests, turnaround time, and the interaction also impact CDx development.

In the case of complementary diagnostics, companion diagnostics restrict patients to receive co-developed therapies based on the outcome emphasizing the biomarker.

### CDx with Alternate Therapies for Infectious Diseases

Antibacterial mAbs are making a comeback, as is phage therapy [153]. With the FDA approving four mAbs for pathogens, the key factor influencing its development in CDx is finding the optimal targets for the pathogen and isotype [78]. Antitoxin antibodies as preventive strategy or as an adjunctive to antibiotics have yielded success [154,155] Motley, Banerjee et al., 2019). Other antibodies targeting the surface proteins–outer membrane proteins involved in adhesion, immune evasion, and bacterial biosynthesis may be strategized for CDx biomarker development [156,157,158,159].

Polysaccharides, namely, lipopolysaccharide (LPS) and capsular polysaccharide (CPS) are popular targets. CPS-targeted antibodies improve opsonophagocytosis [160]. A successful mAb therapy against a polysaccharide antigen shifts bacterial populations away from utilizing that antigen [161] and, hence, may be utilized in identification with IHC or a fluorescence-activated cell sorter (FACS). Natural LPS antibodies have a high frequency of somatic hypermutations in IgM and IgA against certain glycan signatures that significantly improve the affinity, specificity, and stability of several preclinical antibodies within an application of adding multi-specific binding properties [162].

Three anti-bacterial mAb products based on neutralization of exotoxins have been approved for human use—raxibacumab (ABthrax^®^), obiltoxaximab (Anthim^®^) as a prophylactic for anthrax, and bezlotoxumab (ZINPLAVA^TM^) for recurrent infection by *Clostridium difficile*. Antibiotics might control the bacterial infection but fail to clear released toxins from the bloodstream. The detection of toxins in the blood may be neutralized with raxibacumab, the anti-PA recombinant, a IgG1λ mAb, and prevent disease progression. Serodiagnostic assays for anthrax are based on the detection of antibodies against PA or lethal factor (LF) [163,164], which develop post-infection. However, early detection by sandwich ELISA 24 or surface plasmon resonance 8 could provide a timely diagnosis. Hence, either of the detection methods along with raxibacumab (ABthrax^®^) or obiltoxaximab (Anthim^®^) may be prescribed as a CDx for anthrax.

Known as host defense peptides (HDP), AMPs exhibit antimicrobial activity on both Gram-negative and Gram-positive bacteria, and belong to two main families—the defensins and cathelicidins. Antimicrobial peptides are small peptides (4–50 amino acid residues) with amphipathic conformation [68,83]. The protective effect of AMP against infections has clinical correlations [165,166]. In patients with impaired epithelial AMP production, as in the case of atopic dermatitis, susceptibility to secondary infections is higher in contrast to conditions with increased AMP production (e.g., psoriasis) [167]. Hence, assessment of AMP induction by IHC, Western blot, and RT-PCR may be employed in the diagnostic part of AMP CDx.

As an alternate measure, AMPs have significant efficacy in preventing biofilm formation, despite the heterogenous nature and complexity of biofilm [29,168]. AMP activity on biofilm is best described by growing in multi-well plates or the Calgary device. Fulfilling the requirements for a CDx and the criterion of an in vitro diagnostics IVD, the Calgary device, a flow-cell device, may be combined with an AMP assay [117]. In the case of membrane-active AMPs, membrane permeabilization has an effect, although fluorescence microscopy with fluorophores are used, and an inexpensive method is the microtiter plates for detection with crystal violet [169].

Phage therapies are unlikely be the first-line treatment but can be an alternative in cases that have failed with antibiotic treatments [170]. Phage preparations can be formulated if the preliminary pathogenic profile is known. Both phages and bacteria are subject to continuous co-evolution [171]. Phage therapy has emerged as a potential alternative with success, and one that meets the One Health Approach with the European Green Deal [172]. However, complicating regulatory issues and safety concerns prevent phage use in therapeutics [173]. However, the use of phages with antibiotics is a superior strategy for controlling bacterial pathogens, with a dual approach of stronger bacterial suppression and the reduced capacity for developing phage and/or antibiotic resistance [69]. Phage productivity and phage-mediated bacterial lysis with PAS is beneficial for some phage/antibiotic combinations, but ineffective in others [174,175,176]. A combined approach restores antibiotic sensitivity (Chan et al., 2016). The diverse properties are not exploited in phage–antibiotic combinations. Depending on the magnitude of bacterial suppression, the interactions are categorized as true synergism, additive effects, or as facilitation [67].

Recently, a phage susceptibility test has been developed to simultaneously test hundreds of phages selected from the adaptive phage therapeutic (APT) phage bank against bacteria isolated from a patient. The PST identifies one or more phage for treatment. The phage library, the APT’s phage bank, has been deployed with a companion diagnostic to achieve rapid response and cost-effective therapy for otherwise recalcitrant bacterial infections. Apart from natural phages, synthetic phages with engineered genes can be employed. Minimal phage cassis can replicate well in a wide range of target bacteria. Hybrid phages have interchangeable tails, with lower percentages of homology, and are adaptable to target bacteria. For selectivity, this is combined with the receptor-binding proteins (RBPs) selected by in vitro evolution in the lab, and determines the strain to be killed by the synthetic phage. Multivalent phages with multiple RBPs can expand the host range, if the therapeutic phage is intended to be used on a wider range of bacteria.

With regards to aptamers, recognition and specific binding are promising aspects. The selection of aptamers from oligonucleotide pools by SELEX can allow for a wide range o+{[[/as well as in clinical samples (Qiao, Deng et al., 2018) [58].

With respect to the assay, different formats of detection with aptamers are the enzyme-linked oligonucleotide assay (ELONA), fluorescence-based assays, aptamer-based flow cytometry, fluorogenic assays, and electrochemical sensing, which are promising diagnostic tools. The next criterion in CDx, which is the biomarker development, can be accomplished by the highly multiplexed slow off-rate modified aptamer (SOMAmer)-based biomarker discovery. The diagnostic platform SOMAscan detects and quantifies >1300 proteins simultaneously in a variety of clinical samples [177]. In one instance, SOMAscan discovered several biomarkers [178].

The other tool is clustered repetitive interspaced short palindromic repeats, CRISPR-associated enzyme (CRISPR–CAS), which is used against antimicrobial-resistant pathogens. Diagnostic platforms use Cas enzymes (Cas12/Cas13) incubated with the target nucleic acid and fluorescent ssDNA/ssRNA reporters. On detection of the target, the Cas enzymes trans-cleave and generate a robust fluorescent signal that has been correlated with PCR-based methods [179]. A different multiplexing strategy also uses Cas9 to enrich low-abundance targets from complex backgrounds before NGS. This method aided in distinguishing Klebsiella pneumoniae carbapenemase (KPC) and New Delhi metallo-β-lactamase (NDM) from five clinical isolates of K. pneumoniae [179] (Gootenberg et al., 2017)

CRISPR–Cas9, Cas3, and Cas13 are the potent sequence-specific antimicrobial enzymes. CRISPR interference (CRISPRi) uses catalytically inactive Cas9 (dCas9) and single-guide RNA (sgRNA) to repress sequence-specific genes [180]. Cell death occurs when single-guide RNA is directed to genes on the chromosome or plasmids containing a toxin–antitoxin system. In the absence of toxin–antitoxin, plasmid clearance or a drastic reduction in copy number is achieved [181,182,183].

Recently, recognition of surface proteins on methicillin-resistant staphylococcus aureus (MRSA) strains by aptamer and CRISPR–Cas12a-assisted rolling circle amplification was achieved [184]. There is an arcade of CRISPR–Cas/aptamer combinations and target bacteria to be tested. CRISPR–Cas and aptamers can be combined to treat and/or diagnose resistant bacterial infections due to their aforementioned characteristics, making a pair with a companion test. Exogenous short interfering RNA (siRNA) alters gene expression but exhibit high stability with minimal toxicity, modulating virulence, drug resistance, and pathogenesis. The siRNA–aptamer conjugates increased therapeutic efficacy and safety [52]. As a result, a companion test may include an siRNA–aptamer combination. 

## 8. Conclusions

Resistance persists despite antibiotic drug development. With increasing failure rates in new antibiotics, focus is shifted to alternate antimicrobial therapeutics, namely, monoclonal antibodies, antimicrobial peptides, aptamers, and phage therapy. A multi- ‘omics’ approach validates these compounds as drug targets. With a supportive background, these alternative therapeutics may be tuned with CDx for a targeted therapy. As a diagnostic assay measures the therapeutic target or a gene mutation, it is often linked to a biomarker. In non-oncological conditions, particularly in infectious disease, this is not clinically feasible due to the heterogeneity. No common somatic or genetic variations have been identified, so identifying a subgroup that is likely to respond to a therapeutic approach is essential. Also, the timing of treatment is a major factor in determining the clinical efficacy of a therapeutic group, and this is usually reliant on diagnosis. The likelihood of a broad use of specific tests based on utility across a drug class and more than one relevant biomarker rather than a single molecular entity or analyte needs to be improved. To successfully use alternate therapies, good harnessing of CDx is the key. 

## Figures and Tables

**Table 1:** Comparison of alternate antimicrobial therapies.

Antimicrobial Therapy	Anti-microbial Spectrum	Mode of Action	Immune Response	Risk of Developing Resistance	Cross Resistance	Synergic Effects	Toxicity
Monoclonal Antibody [78]	Narrow	Non-Bactericidal	Specific	Low	No	Yes	Mild
Antimicrobial peptides [35]	Broad	Bactericidal	Non specific	Low	No	Yes	Moderate
Aptamers [79]	Broad	Bacteriostatic	None	Low	No	Yes	No
Phages [80]	Narrow/ Broad	Bactericidal	Specific	High	No	Yes	No

**Table 2:** Potential CDx tests for alternate therapies.

Alternate Therapy	Target	Potential Companion Tests
Monoclonal Antibody	Surface proteins— Adhesion—outer membrane proteins Immune evasion Bacterial biosynthesis	Immunohistochemistry Western blot Fluorescence Activated Cell Sorter Enzyme Linked Immuno Sorbent Assay Surface Plasmon Resonance
Antimicrobial peptide	Non specific	Immunohistochemistry Western blot Real Time-Polymerase Chain Reaction
Aptamers	Antisense oligonucleotide Gene silencing	CRISPR-Cas ((clustered regularly interspaced short palindromic repeats–CRISPR associated) Aptamer Linked Immobilized Sorbent Assay Enzyme-Linked Oligonucleotide Assay Fluorescence-based assay, aptamer-based flow cytometry
Phage therapy	Antibiotic sensitivity	Phage bank

## Data Availability

Data sharing not applicable.
